# Effects of a Novel Mouthwash on Dental Remineralization

**DOI:** 10.4172/2161-1122.1000432

**Published:** 2017-05-05

**Authors:** Janet Ajdaharian, Thair Takesh, Afarin Anbarani, Jessica Ho, Petra Wilder-Smith

**Affiliations:** Beckman Laser Institute, University of California, Irvine, USA

**Keywords:** Dentine sensitivity, Mouthwash, Enamel Microhardness, Demineralization

## Abstract

**Objective:**

The goal of this study was to evaluate the *in vivo* effects of a novel mouthwash on enamel remineralization.

**Materials and Methods:**

Ten healthy volunteers wore removable intra-oral appliances for three study arms with duration of 5 days each. In 1 study arm, subjects used Oral Essentials Sensitivity Formula^R^ mouthwash; in another arm they used Sensodyne^R^ mouthwash, and in the third arm they used no mouthwash at all. Sequence of mouthwash use was randomized, and study participants and researchers were blinded throughout the study. Subjects used Crest Total Care^R^ toothpaste throughout the study. During a one week washout period before study begin and between each study arm, subjects also used Crest Total Care^R^ toothpaste. A total of 300 enamel samples were included in this study, 150 served as baseline controls, and 150 as test samples subjected to demineralization prior to intra-oral wear. At the end of each study arm, enamel chips were removed from the appliance and underwent standard Microhardness (Knoop) measurements, as did the control samples. Enamel microhardness in the test vs the 2 control groups was compared using the Kruskal-Wallis one-way analysis of variance with post-hoc Tukey’s test to test for differences in remineralization between the 3 treatments.

**Results:**

Both mouthwashes demonstrated similar levels of recovery from demineralization as the “no mouthwash” arm of the study, with no significant differences for all groupings and comparisons (p>0.05).

**Conclusion:**

A novel mouthwash for sensitive teeth supports enamel recovery from demineralization.

## Introduction

Based on a variety of factors such as saliva composition and production rate, intake of food and drinks, and oral biofilm composition, the pH on the tooth surface is in constant flux throughout the day. The enamel surface is directly affected by these pH levels, with the tooth surface undergoing closely linked cycles of de-and remineralization. The reduced enamel surface hardness that results from demineralization [1–3] is paralleled by a heightened risk of abrasion and attrition [4–6]. Variables that affect the rate of demineralization include the pH and duration of the acid challenge [3–5,7–9]. Prior to actual tissue loss, surface remineralization can occur through the replacement of lost mineral ions, typically from the salivary reservoir of calcium and phosphate ions, and the dental biofilm may also harbor mineral ions that play a role in this process [3–11]. Mouthwashes and toothpastes can be helpful in supporting dental recovery by promoting remineralization after acid attack [12–14].

The goal of this study was evaluate the *in vivo* effects of a novel formulation on enamel recovery from demineralization as measured using standard Knoop microhardness testing. This study was designed as a double-blinded, randomized study, wherein neither subjects, clinicians, microhardness testers, nor any other members of the study were aware of product allocation or treatment/control status of the enamel chip samples.

## Materials and Methods

This study was performed in full compliance with the treatment guidelines provided in the Helsinki Accords for Human Research, and with UCI IRB protocol 2013-9778. All subjects signed an informed consent form prior to enrollment in this study. Subjects consisted of 10 healthy volunteers (7 females and 3 males), age 18–45, each with a minimum of 16 clinically and radiographically healthy teeth as defined by clinical examination, and with an absence of any apparent pathology.

### Overview

A total of 300 enamel samples were included in this study, 150 of which served as baseline controls, and 150 as test samples for intra-oral wear. Ten subjects wore custom fabricated intra-oral retainers for 3 study arms of 5 days each, with 5 sterilized enamel chips attached to the palatal area of the retainer. New chips were used for each arm of the study. The study had 3 arms: in one arm subjects used no mouthwash; in another arm subjects used Oral Essentials Sensitivity Formula^R^ (Oral Essentials, Beverly Hills, CA 90210) mouthwash, and in the third arm they used Sensodyne^R^ mouthwash (GSK, Warren, NJ 07059). During the one week washout period before the first arm and between each arm of the study subjects also used Crest Total Care^R^ toothpaste (P & G, Cincinnati, OH 45224) toothpaste. Subjects were supplied with a new Oral B^R^ (GSK, Warren, NJ 07059) toothbrush at the beginning of each new arm of the study. The sequence of mouthwash use by the subjects was randomized.

Data was collected for the following time points:

- Day 0: baseline
- Day 5: No mouthwash used (negative control)
- Day 12: One week washout completed
- Day 17: Use of first mouthwash completed
- Day 22: One week washout completed
- Day 27: Use of second mouthwash completed

### Clinical protocol

Standard alginate impressions of the upper jaw were recorded in all subjects. This was repeated prior to each of the 3 arms of the study. The impression was used to fabricate a customized removable appliance designed to hold five enamel blocks in five standard locations. A new retainer was fabricated for each arm of the study. Retainer fit and comfort were checked prior to attaching the enamel chips to the retainer. During each arm of the study, subjects brushed their teeth for 2 minutes twice daily and abstained from all oral hygiene measures other than the prescribed protocol. The mouthwashes were all dispensed in the same generic nontransparent containers. Mouthwash use proceeded as follows: with the retainer in place the subject rinsed with the standard, recommended amount of mouthwash around the palatal area of the appliance where the chips were mounted for 60 seconds. Neither the appliance nor the enamel specimens were brushed. Following expectoration, the subjects did not rinse, drink or eat for 30 minutes. During the duration of the study, the subjects were instructed not to use any products that were not provided by the study staff including but not limited to floss and baking soda. Subjects wore the retainer for a minimum of 22 hours per day, removing it during meals and placing it in a sealed container during this time. Subjects recorded appliance wear every evening on a time log to monitor compliance.

### Samples

**Figure 1:**
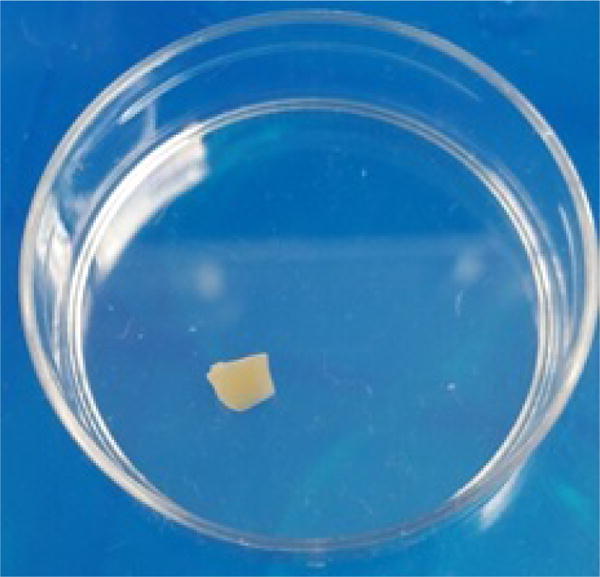
Sterilized enamel chip ready for mounting on retainer.

**Figure 2:**
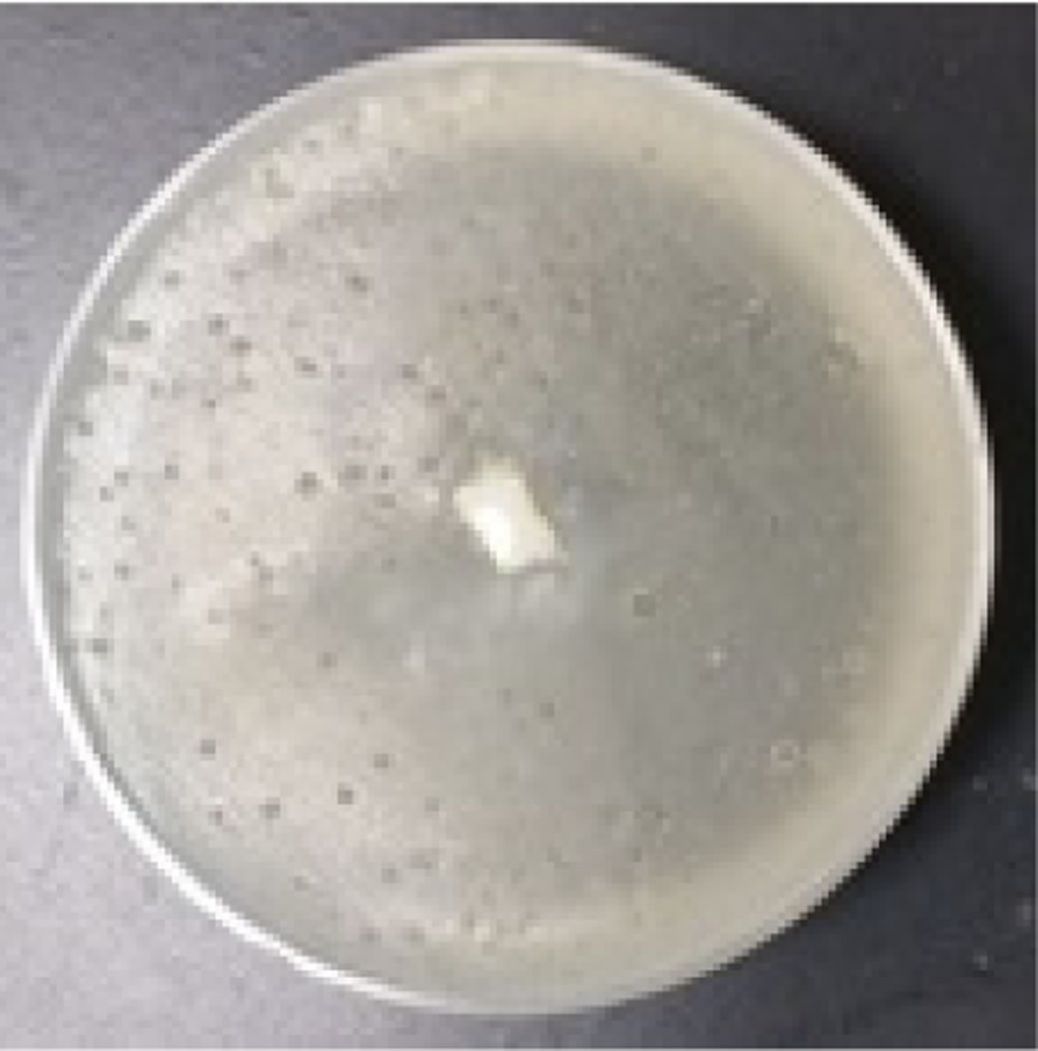
Enamel chip embedded for microhardness testing.

From sterilized extracted teeth classified as healthy by an experienced dentist (28 years of dental practice) using a loupe and headlamp, 2 enamel chips 4 mm × 4 mm × 3 mm were cut from the same area of each extracted tooth (Figure 1). A total of 300 chips were prepared in this fashion. From each chip “pair”, one chip was held back as a control sample, and underwent standard Knoop microhardness testing (Figure 2), an established and standard technique for measuring enamel mineralization [15]. These samples were then stored per standard protocol in de-mineralized water at a temperature of 4°C and 100% humidity, and protected from ambient light in a sealed and labeled double-walled container. A total of 150 control samples were evaluated in this way.

The remaining 150 chips were subjected to 6 hours of demineralization using an acetate/calcium/phosphate buffer at pH 4.4. The buffer contained: 2.0 mmol/l calcium, 2.0 mmol/l phosphate and 75 mmol/l acetate, with 40 ml per sample used individually [16].

The demineralized chips were then attached to the palatal surface of each retainer with approximately 0.75 to 1.5 cm separation between them and left in place over the duration of that arm of the study (5 days). At the end of each arm, samples were detached from the retainer for microhardness measurements consisting of 3 individual microhardness indentation measurements in Knoop units (Figure 2). Microhardness (Knoop) data points were collected using indentation measurements at 3 locations per sample: one in an area of typical appearance, one in an area with the healthiest (best) appearance, and one in an area with the most damaged (worst) appearance.

### Data analysis

The Kruskal-Wallis one-way analysis of variance with post-hoc Tukey’s test was used to test for differences in microhardness between the 3 treatments.

## Results

Microhardness results per sample and treatment are shown below in Table 1. All tooth samples underwent statistically similar levels of de-and remineralization, softening by a mean 27.24% (S.D. 4.8%) due to demineralization and then hardening again after 5 days of intra-oral wear to approximately the pre-demineralization level (p>0.05) and demonstrating no significant differences for all groupings and comparisons. Thus, no significant differences were determined between the levels of re-hardening after use of either of the mouthwashes or no mouthwash at all (p>0.05).

## Discussion

The goal of this project was to evaluate the *in vivo* effects of a novel mouthwash on enamel recovery after demineralization and 5 days of intra-oral exposure. Samples were eroded by means of a standard technique through exposure to demineralization using an acetate/calcium/phosphate buffer [16]. The technique was developed by the Featherstone laboratory and has been used as standard procedure for many years. In order to permit ex vivo microhardness measurements on enamel subjected to intra-oral conditions, pre-eroded enamel slabs were attached onto a removable retainer. Then the mouthwash was rinsed around the samples while the retainer was in place. Although this approach has been used for many years, the enamel slabs are not exactly comparable to the enamel on in situ teeth, due to potential differences in adsorbed components, as well as biofilm. Moreover, it would be helpful if diet were controlled in future studies, to remove an additional potential source of variability. Thus, additional larger controlled studies over longer periods of time are needed to more closely evaluate product effects after mid- and long-term clinical use.

This pilot study demonstrated that a novel mouthwash supports enamel remineralization at a similar level as an existing mouthwash for sensitive teeth. Mouthwash alone does not adequately maintain oral health, and is typically seen as an adjunct to adequate physical brushing with toothpaste and flossing. However, its use can enhance oral hygiene and also help to mitigate symptoms of dentinal sensitivity.

In this study, demineralized enamel chips regained their original microhardness equally in all 3 arms of the study. This finding suggests that remineralization process was similar regardless of the type of mouthwash used or even use of no mouthwash at all. This is interesting, because the test formulation contains a high concentration of minerals, but no fluoride, whereas the control formulation does contain fluoride. Perhaps this observation can be attributed to the remineralizing action of the fluoride toothpaste that was used throughout the study.

## Conclusion

In conclusion, this study determined that a novel sensitive mouthwash formula achieves comparable remineralization of enamel as one of the leading dentinal sensitivity mouthwashes. Additional, larger studies are needed to ascertain the effects of this novel mouthwash on *in vivo* enamel demineralization, and on specific categories of dentinal sensitivity.

## Figures and Tables

**Table 1:** Comparison of mouthwash and control microhardness ratio.

No Mouthwash (Control)	Oral Essentials Sensitivity Formula Mouthwash	Sensodyne Mouthwash (Active Control)
Mean Microhardness (MH) ratio: Mean Final/Original MH	Mean Microhardness (MH) ratio: Mean Final/Original MH	Mean Microhardness (MH) ratio: Mean Final/Original MH
1.05 (S.D.= 0.19)	1.12 (S.D.= 0.18)	1.08 (S.D.= 0.13)
